# Priorities in prevention and control of flood hazards in Iran 2019 massive flood

**Published:** 2019-04

**Authors:** Laleh Sharifi, Saied Bokaie

**Affiliations:** 1 Uro-Oncology Research Center, Tehran University of Medical Sciences, Tehran, Iran; 2 Department of Epidemiology, Faculty of Veterinary Medicine, University of Tehran, Tehran, Iran

**Keywords:** Global climate change, Flood, Communicable diseases

## Abstract

Global climate change leads to an increasing in the number and severity of weather events such as floods. Floods have been reported one-half of all weather-associated disasters with high impacts on countries. Global warming causes a different pattern of rainfall in Iran caused long-term drought since 30 years ago and recent heavy raining which lead to a massive flood in this country. It is predicted that health subsequences of Iran 2019 flood such as communicable diseases vary due to the geographical extent and different climates of flooded areas. However, observing long term and short term preventive measures can be effective to reduce the high impact of flood in Iran.

## INTRODUCTION

Global climate change, deforestation, rising sea levels, and population growth lead to an increasing in the number and severity of weather events such as floods. Floods have been reported one-half of all weather-associated disasters, have affected 2.3 billion people and responsible for 157,000 deaths during 1995–2015 worldwide (1). It has been predicted that the number of threatened people may reach to 2 billion in 2050 (2). Floods impose a high economic burden near to 60 billion USD annually, mostly in developing countries (3).

Asia is at more risk of flood compared to other parts of the world. Annually about 400 million people are at direct exposure to floods in Asia and the number of flood-related lives lost in this area is near to 93% of all flood-related deaths all around the world (4). The risk class of Iran for natural events is 8 out of 10. In the previous four decades in Iran, natural disasters have caused more than 109,000 deaths and 150,000 injuries (5).

Global warming causes a different pattern of rainfall in Iran as in other parts of the world. Due to the climatic changes, Iran has experienced a long-term drought since nearly 30 years ago. Many ecosystems have changed, many parts of forests have disappeared and lands diminished, rivers and lakes dried up during this period. People have moved and settled in areas where used to be the river banks and beds before the drought. These alterations lead to a catastrophic flood in Iran. Almost all 31 provinces of Iran had heavy rains that in lacking trees and bushes washed away the unprotected ground (6). Based on the Iranian Red Crescent report more than 2000 cities were affected by flash floods. The overwhelming flood had an impact on 10 million Iranian people in some ways. At least 78 deaths have been reported and the number of injuries reached to 1136 cases and 500,000 people had to displace their houses permanently or temporarily (7). Iran's flood has been accompanied by snake attacks in the north of the country and locusts attacking the south. Financial losses have been calculated around 8 billion USD because of the enormous destruction of roads, houses, infrastructures, agricultural lands, and animal farms (6).

In this paper we aimed to review the health related subsequences of floods and give disease prevention clues in regards to Iran 2019 vast floods.

### Flood-related health concerns.

Floods affect people health by different ways. The health-related influences of floods are varied and are associated with a set of factors (8–10). Flood consequences are categorized based on the time after the event:

1- The early phase is associated to first problems during or just after flooding such as injuries, drowning, hypothermia (particularly in children), animal bites, acute asthma, skin rashes and clusters, outbreaks of gastroenteritis, and respiratory infections epidemics (11–13).
2- Inter-mediate phase associated to middle-term health problems including infected wounds, infections, zoonosis, poisoning, mental problems, and starvation (9).
3- Late phase is associated with long-term health problems including disability, chronic disease, and poverty associated malnutrition. It is notable that flood victims are exposed to both physical and social-emotional, and health-mental problems. Victims have to immigrate to other places, cause the risk of solastalgia with high psychological distress such as anxiety, depression, irritability, and sleeplessness (9, 14–16). Long duration floods lead to severe health problems for people, especially for elderly and disabled people who live in unhygienic and poor condition (17). In long term, natural disasters are also associated with onset and/or development of cancer due to disruption of cancer treatment systems especially for older adults (18, 19).

After the flood subsidence, people will be exposed to many health problems in the condition that the injured people are still mourning the loss of their family members and homes. The floods have serious subsequences due to contamination of drinking water and accumulation of wastes. Standing waters after the flood became an active center for the proliferation of pathogen agents and vectors (20, 21). Population displacement (22), overcrowding and close contact among refugees in low hygienic conditions increase the risk of outbreaks of infectious diseases (23, 24). Flooded environments are predisposed to infectious diseases including water-borne, vector-borne and rodent-borne diseases (25).

### Water-borne diseases.

Contamination of drinking water systems with floods is the leading source for water-borne diseases after heavy rainfalls and floods. Floods carry bacteria, parasites, and viruses into the drinking water supplies (26). The epidemics of waterborne diseases around the world have been reported concurrent to flood events from 1980 to 2006 (26–28).

There are large data about outbreaks of cholera, nonspecific diarrhea, cryptosporidiosis, rotavirus, typhoid and paratyphoid, and hepatitis A with floods (29–31). However there is an increased risk for infections by direct contact with contaminated waters such as dermatitis, conjunctivitis, wound infections, and ear, nose, and throat (ENT) infections but fortunately these diseases are not predisposed to develop epidemic outbreaks (30).

### Vector-borne disease.

Rainfall events have been shown to influence arthropod vectors by changes in their production, growth, behavior, and population dynamics as well as their pathogens and reservoirs (32). Increasing in rainfall amounts and stagnant waters lead to the creature of new breeding grounds for mosquitoes such as phlebotomus, *Aedes* and *Anopheles* genus and consequently an explosive epidemic of Mosquito-borne disease can be predictable (26, 33).

The close link between periods of heavy rainfalls and resultant flooding and vector-borne diseases such as leishmaniasis, malaria, Rift Valley fever, yellow fever, dengue and dengue hemorrhagic fever, and West Nile fever has been well understood (30, 34–36).

Vector control methods are based on making the environment unfavorable for the survival, development, and reproduction of the vector. In the case of mosquito control, use of insecticide and indoor spraying lead to decrease in the spread of malaria and other mosquito-borne diseases (23).

### Rodent-borne diseases.

Rodent transmitted diseases increase during periods of heavy rains and flooding. There are multitude of reports about outbreaks of leptospirosis and Hantavirus Pulmonary Syndrome after flood events worldwide (35). Wild grasses growth are reinforced during heavy rains and flooding subsequently supporting rodent population expansion. Also, flood force rodents to leave their burrows and find new environments close to humans. Disease transmission occurs via contact of the skin and mucosal membranes with rodent urine contaminated water or mud (37). Discourage rodent breeding is vital for controlling the rodent-borne diseases by the collection of wastes and suitable disposal of them as well as clearance of tall grasses around inhabited places (38).

### Preventive measures.

The results of a study on some Asian countries for a period of 2005–2017 showed that natural disasters such as flood mainly upsurge migration rate, price level, increase health costs, high energy request and poverty incidence, which negatively affect financial resources and low economic growth (39). These findings underscore the importance of the development of high qualified preventive methods to decrease the burden of disastrous flooding. Preventive strategies include long term and short term measures.

### Long term measures.

Long term issues for flood prevention include legislation, administrative and technical issues. Governments should improve flood surveillance on a local, national, international level and create disaster-preparedness programs and early warning systems. Health administrative should enforce high standards of hygiene, promote tap-water quality regulation and monitoring, and keep communicable disease control systems active and effectual (30).

The continuous monitoring of hospitals and other relief centers is one of the major measures in the prevention of disasters. Structural and nonstructural preparedness of hospitals, as the most vital place providing health services and a safe place for evacuation, is an important issue of public readiness against flood. It would be operative to retrofit hospital buildings every six months and compile written tactics for administrative procedures during catastrophes. Hospital managers should pass essential courses about disaster management and should promote educational programs for hospital workers to ensure their knowledge and skills during a flood event. Furthermore, all the hospitals are required to have equipped with an Emergency Operations Center (EOC) with a regular checking system. Until now many researches have been carried out about the disaster readiness of hospitals in cities of Iran (40–43), but the results of a study on 224 hospitals safety showed that the mean score of 3 safety components including structural, non-structural and functional capacity was low (32.4 out of 100) (5). In addition to the need of increasing the quality of hospitals in dealing with such disasters as floods, it is necessary to pay more attention to remote areas of the country that have limited access to preventive facilities.

It is necessary to make sure that people who are at risk of flood efficiently acquire adaptive behaviors that are proposed by public health officials to protect themselves against flooding threat. Public educations through media and local agents should be performed for developing preventive behaviors during flood alert, after the alert for flood (requiring or not requiring evacuation), and post-flood periods (44).

### Short-term measures.

Observing the flowing recommendation after happening the flood by disaster administrators and people can significantly reduce the hazard of contagious diseases after flood events.

### Providing clean drinking water by chlorination.

Chlorination of water is the leading preventive method to reduce the risk of water-borne infections. Free chlorine is effective against almost all water-borne pathogens. The most appropriate forms of free chlorine for household are liquid sodium hypochlorite, solid calcium hypochlorite, and bleaching powder. The amount of chlorine has to be detected for each situation based on the concentration of organic material of water. It is important that the residual concentration of chlorine should reach to 0.2–0.5 mg/l after 30 minutes, which can be detected by a test kit (27).

### Health education.

Providing post-flood educational packages promoting good personal hygienic practice are of high importance. Media-based programs or informative material about boiling or chlorination water, safe food preparation techniques, and early diagnosis and treatment of flood-associated diseases can reduce the burden of health problem (27).

### Vaccination.

Outbreaks of anthrax may occur after the floods due to percolating the spores buried in soil toward the surface of pasturages (45–47). To control outbreaks of anthrax, vaccination of animals should be considered by officials of the veterinary organizations.

The use of hepatitis A vaccines after flood are recommended for high-risk groups but mass immunization is not recommended (27).

## CONCLUSION

Climate change leads to an increase in number and severity flooding, which is the most common and deadly catastrophe globally. Floods play a significant role in the epidemics of infections because of developing multiplication condition for pathogens and vectors. It is predicted that health subsequences of Iran 2019 flood vary due to the geographical extent and different climates of flooded areas. In addition to outbreaks of communicable diseases and the possibility of strengthening the endemic diseases new health problems will occur due to climate changes such as snake attacks in the north of the country and locusts attacking the south (7). During days after flooding, a quick disease risk assessment should be carried out by health officials in order to identify the proper interventions and medical needs.
